# Obstructive Jaundice in Chronic Pancreatitis

**DOI:** 10.1155/1989/75793

**Published:** 1989

**Authors:** M. J. Hollands, J. M. Little

**Affiliations:** Department of Surgery Westmead Hospital Westmead 2145 NSW Australia

## Abstract

Significant obstructive jaundice in chronic pancreatitis is generally considered to be rare. Eleven of 57 consecutive patients with proven chronic pancreatitis have developed significant obstructive jaundice of more than transient duration. Eight presented as jaundice complicating known pancreatitis and three as jaundice of unknown cause.

Life table analysis showed a steady rise in the risk of developing jaundice up to the end of 10 years from the onset of chronic pancreatitis. Jaundice was found to occur in the presence of more “destructive” disease, and jaundiced patients had a higher incidence of pancreatic calcification, diabetes and malabsorption at the time of presentation with jaundice.

Obstructive jaundice caused by chronic pancreatitis was found to carry a good prognosis for jaundice, for pain and for life. Only one of the 11 patients died in hospital.

It is important to distinguish chronic pancreatitis from cancer in these patients. Pre-operative and intraoperative cytology have been helpful. Stent insertion is not an appropriate method of treatment for these patients because of the benign nature of the disease and the possibility of exacerbating the pancreatitis. It is important to be aware of another form of “malignant masquerade” causing obstructive jaundice.


*HPB Surgery* 1989, Vol. 1, pp. 263–270
Reprints available directly from the publisher
Photocopying permitted by license only

© 1989 Harwood Academic Publishers GmbH
Printed in Great Britain

### OBSTRUCTIVE JAUNDICE IN CHRONIC PANCREATITIS

M. J. HOLLANDS and J. M. LITTLE

*Department of Surgery, Westmead Hospital, Westmead 2145 NSW*

*(Received 24 May 1988)*

Significant obstructive jaundice in chronic pancreatitis is generally considered to be rare. Eleven of 57 consecutive patients with proven chronic pancreatitis have developed significant obstructive jaundice of more than transient duration. Eight presented as jaundice complicating known pancreatitis and three as jaundice of unknown cause.

Life table analysis showed a steady rise in the risk of developing jaundice up to the end of 10 years from the onset of chronic pancreatitis. Jaundice was found to occur in the presence of more "destructive" disease, and jaundiced patients had a higher incidence of pancreatic calcification, diabetes and malabsorption at the time of presentation with jaundice.

Obstructive jaundice caused by chronic pancreatitis was found to carry a good prognosis for jaundice, for pain and for life. Only one of the 11 patients died in hospital.

It is important to distinguish chronic pancreatitis from cancer in these patients. Pre-operative and intra-operative cytology have been helpful. Stent insertion is not an appropriate method of treatment for these patients because of the benign nature of the disease and the possibility of exacerbating the pancreatitis. It is important to be aware of another form of "malignant masquerade" causing obstructive jaundice.

KEY WORDS: Obstructive jaundice; chronic pancreatitis; surgical treatment.

### INTRODUCTION

Approximately one third of patients with chronic pancreatitis develop jaundice[1]. It may be due to hepatocellular disease or due to biliary obstruction. Abnormal liver function tests and transient rises in bilirubin are reasonably common during acute exacerbations of chronic pancreatitis[2]. In Bradley and Salam's large series of 868 patients, 125 (14%) had jaundice. In 29 of the 125 jaundiced patients (22%), the jaundice was due to extrahepatic obstruction. In only 13 patients (1.5% of the total series) was obstruction due to benign pancreatic disease. In the other 16 patients the jaundice was secondary to gallstone disease[3]. In many patients stenosis of the intrapancreatic portion of the common bile duct is clinically silent[3,4,5]. In others there is an elevated alkaline phosphatase (S.A.P.) and this may be the earliest finding in patients with biliary obstruction[2,5,6,7,8]. In patients with incomplete biliary obstruction biliary cirrhosis may supervene[4,9]. Lasting, clinically significant obstructive jaundice is much less common being seen in less than 4% of cases in most series[1,3,10,11].

Segal *et al.*[9] have described three clinical types of chronic pancreatitis with biliary obstruction — transient, recurrent, and persistent. The present paper is concerned solely with clinically significant and lasting jaundice, not with the transient variety. Obstructive jaundice may present as a progressive problem. Sarle *et al.*[1] emphasised that anatomical changes in the common bile duct in patients with pancreatitis are common but symptoms are rare. Obstructive jaundice may present as a progressive problem developing against the background of known pancreatitis. At other times, it may present as relatively painless jaundice, making it difficult to distinguish from the jaundice caused by cancer.

Just as there may be confusion with cancer, so there may be confusion between the relative roles of chronic pancreatitis and biliary duct obstruction in causing pain. Recently, routine biliary bypass has been advocated as part of a conservative surgical program designed to preserve pancreatic function[12]. The results of such methods of treatment are not yet documented.

Finally, there is uncertainty about the factors which contribute to the development of jaundice, and there is no clear patient profile which may alert a clinician to anticipate incipient jaundice or to diagnose pancreatitis when the jaundice is established.

### MATERIALS AND METHODS

Patients presenting with proven chronic pancreatitis to one surgical unit between 1979 and 1986 have been included. All patients have been followed for at least two years in order to exclude as far as possible those with undiagnosed malignancy. Only patients with clinically significant and progressive obstructive jaundice caused by pancreatic pathology and present for more than one week have been included. Diagnosis of chronic pancreatitis was confirmed at autopsy in one patient. The remainder were required to satisfy at least two of the following criteria: a typical history of upper abdominal pain; documented rises in serum amylase; calcification in the pancreas demonstrated on conventional radiology or computerised tomography; an endoscopic retrograde cholangiopancreaticogram (ERCP) demonstrating ductal irregularity and filling of the secondary ducts, dilatation of pancreatic ducts or pancreatic calculi; and biopsy or cytological absence of malignancy. Patients with jaundice caused by gallstones were excluded from the study. All patients have been personally followed by one of the authors (JML).

Data has been gathered prospectively and maintained on a computerised database. Statistics were calculated using a Stats plus programme. Non-parametric analysis was used and Fisher's exact test chosen to test the significance of results except where the expected frequency was more than 5 in which case Chi-squared analysis was chosen.

### RESULTS

### *General*

Eleven patients of 57 with proven chronic pancreatitis presented with clinically significant obstructive jaundice lasting more than one week. Seven of the 11 were male, compared to 35 of the remaining 46 patients. The median age of the jaundiced patients was 44 years (range 22–69 years). The median age of the 46 non-jaundiced patients was also 44 years (range 15 to 74 years). Three of the 11 patients presented as jaundice of unknown cause. The remaining 8 were known to have chronic pancreatitis before their jaundice developed. Specific details of liver function and of common bile duct dilatation have been listed in Table 1.

The median duration of symptoms from onset of pain to onset of jaundice was 7

**Table 1** Tests of Liver Function

| *Patient No.* | *Bili* | *SAP* | *ALT* | *GGT* | *CBD diam. (mm)* |
|---|---|---|---|---|---|
| 1 | 90 | 275 | 154 | 138 | 14 |
| 2 | 92 | 360 | 232 | 800 | 15 |
| 3 | 136 | 198 | 150 | 161 | 17 |
| 4 | 430 | 1960 | 176 | 540 | 13 |
| 5 | 163 | 345 | 177 | 400 | 11 |
| 6 | 260 | 420 | 1010 | 1760 | 14 |
| 7 | 117 | 1610 | 75 | 480 | 15 |
| 8 | *6 | 780 | 74 | 1410 | 13 |
| 9 | 146 | 253 | 239 | 229 | + |
| 10 | 464 | 264 | 138 | 464 | 13 |
| 11 | 68 | 316 | 98 | 202 | 14 |

Legend:

* This patient presented with jaundice but was treated by external drainage prior to referral hence the low bilirubin.
+ No measurement was recorded of this patient's dilated CBD.

years in the jaundiced patients (range 1-10 years). The median duration of symptoms for the 46 non-jaundiced cases before presentation to this hospital for treatment was 5 years (range 1-50 years). A Mann-Whitney test shows this difference to be statistically insignificant ($z = -0.65$, $p > 0.5$). Life table analysis showed, however, that the incidence of jaundice increased with time to 10 years from the date of first symptoms (Figure 1). None of the 10 patients with symptoms for longer than 10 years at the time of presentation to this hospital have become jaundiced. The increase in risk appears to be linear, at a rate of about 2.6% of those at risk each year ($R^2 = .9309$, $p < .0001$).

*Aetiology of pancreatitis*

Five of the 11 jaundiced patients were alcoholics, four had idiopathic pancreatitis, one post-traumatic pancreatitis and one familial pancreatitis. Twenty-eight of the 46 non jaundiced patients had alcoholic pancreatitis, 8 idiopathic pancreatitis, 3 pancreatitis following trauma, 2 familial pancreatitis and 5 chronic pancreatitis secondary to biliary disease. There was no example of chronic biliary pancreatitis amongst the jaundiced patients.

*Pancreatic duct size*

Pancreatic ducts were considered to be dilated if >7mm in size, a size determined as being the minimum size suitable for pancreaticojejunostomy. Amongst the 11 jaundiced patients, 2 had small pancreatic ducts, 6 had irregular ducts dilated to less than 7 mm., while 3 had ducts dilated to greater than 7 mm. diameter. Amongst the remaining 46 patients, the ducts status was unknown in 10, small in 11, irregular and slightly dilated in 8 and dilated beyond 7 mm. in 17. There did not appear to be any preponderance of a particular pancreatic duct status amongst the jaundiced patients.

*Glandular calcification*

Radiological calcification was noted in 8 glands amongst the jaundiced patients, while it was present in only 10 of the 46 non-jaundiced patients. This difference is significant (chi-square = 8.452, $.005 > 2p > .001$).

*Diabetes*
Four of the 11 jaundiced patients had diabetes requiring insulin when they presented with jaundice. This was true of only 4 of the 46 who were not jaundiced at first presentation. Again this difference is significant (Fisher exact probability $p = .037$).

*Malabsorption*
Three of the 11 jaundiced patients had malabsorption at the time of presentation with their jaundice that required permanent supplementation with pancreatic enzyme preparations. Only 2 of the remaining 46 patients had malabsorption requiring treatment. This difference is of marginal statistical significance (Fisher exact probability $p = .045$).

*Surgical Treatment*
One patient required no surgical treatment, his jaundice subsiding spontaneously at two weeks. In the remaining 10, the gallbladder was used as a bypass conduit 5 times, and the common hepatic duct 4 times. One patient had a transduodenal sphincterotomy.

The pancreatic pain was independantly treated in 5 patients, 3 times by pancreaticojejunostomy, once by sphincteroplasty and once by total pancreatectomy.

*Hospital Mortality*
There was only one hospital death (within 30 days of surgery or before discharge from hospital at any time). This death occured in a jaundiced patient, who developed haemorrhágic pancreatitis as a complication of his chronic relapsing pancreatitis of alcoholic origin.

*Pain relief*
At the time of writing, 9 of the 10 surviving patients from the jaundiced group were pain free or reported significant improvement in their pain. This compares with 24 of 46 patients improved in the non-jaundiced group. This difference is statistically significant (Fisher exact test $p = .028$).

### DISCUSSION

It is important to recognise that the patients discussed in this paper had proven chronic pancreatitis. Those with obstructive jaundice caused by stones have been excluded, and indeed there was a notable absence of chronic biliary pancreatitis in the jaundiced group. Each patient had clinically significant jaundice that persisted for longer than a week, and all had evidence of duct obstruction with appropriate liver function tests and dilated ducts demonstrated by ultrasound and/or computerised tomography (CT) scanning.

When obstructive jaundice is defined in this fashion, the crude incidence in the 57 patients studied was 19%. This figure is higher than that reported in other series[1,3,10,11]. The cumulative frequency, as determined by life table analysis, reached 34% by the end of the 10th year from the onset of symptoms (Figure 1). Although the numbers are far too small to generalise, the risk seems to diminish at about that time, and none of the 10 patients with symptoms persisting for longer than 10 years have developed obstructive jaundice.

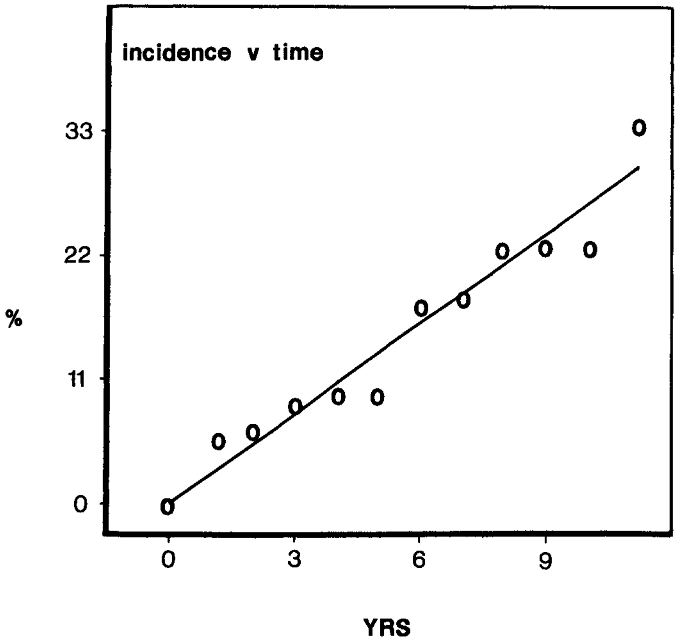

Figure 1

Obstructive jaundice was most commonly seen in those patients with known chronic pancreatitis, and 8 of the 11 in this small study had known chronic pancreatitis for a median of 7 years before jaundice developed. This represents 8 cases occuring in 54 patients with previously known pancreatitis, an incidence of 15% at a median follow-up time of 5 years.

Less commonly, patients presented with obstructive jaundice of unknown cause, and were found to have chronic pancreatitis. This represents a relatively rare syndrome, which was noted only three times in 113 patients presenting with obstructive jaundice as their primary problem during the same period.

There is some evidence from this study and others[12,14] that the disease is more "destructive" in the patients who become jaundiced. There was a higher incidence of diabetes, of glandular calcification and of clinically significant malabsorption amongst the jaundiced patients. Calcification was also very common in the patients described by other authors[1,5,15].

From a clinical point of view, distinction of chronic pancreatitis from cancer is all important[8,11,15,16]. The earliest surgical literature on this subject emphasises this problem[17,18,19,20]. Even after extensive investigation, up to 10% of patients may prove to have unexpected cancers[11,15]. The diagnosis of cancer is particularly important in an era when stenting is frequently employed as a method of palliative treatment for malignant jaundice[21,22]. We have found preoperative and intraoperative cytology of particular value in diagnosing carcinoma. The degree of confidence with which cytology can rule out carcinoma is not yet known. While the specificity of cytological examination for pancreatic cancer approaches 100%, the number of false negative cases is yet to be ascertained accurately, but appears to be about 20%[23,24,25]. Such negative or equivocal results continue to be a problem. We are at present following four patients with negative or equivocal cytology in whom we did not feel that radical resection could be justified since no diagnosis of malignancy could be proven. None of these patients complained of pain.

Only one of the 11 jaundiced patients in this series required no surgical treatment. A number of operations can be used including sphincteroplasty, choledochoduodenostomy, choledochojejunostomy, and cholecystojejunostomy. Littenberg[13] has emphasised that the technical factors are an important consideration in the choice of operation. A small common bile duct represents one potential hazard[26]. Sphincteroplasty should only be used if the stricture is short[8]. Some authors have suggested that utilizing the gallbladder as a form of drainage conduit is associated with an unsatisfactory incidence of cholangitis and the unpredictability of the long term patency of the cystic duct[11,13,27,28]. Bradley and Salam[3], have advocated choledochoduodenostomy because bile is not diverted away from the duodenum, because future pancreatic surgery is not compromised and because stricture from this anastomosis is uncommon. In our experience it seems that the gallbladder works perfectly well as a drainage conduit, as does the common hepatic duct. Pancreatitis must be treated on its merits, and according to the size of the pancreatic duct.

Stenting is not favoured because of the common occurrence of cholangitis and stent blockage[21,22] as well as the theoretical risk of provoking acute pancreatitis from partial pancreatic duct obstruction.

The outcome of treatment in these 11 patients has been remarkably good. Nine of the 10 surviving patients have remained free of jaundice and with their pain improved. One death occured in an alcoholic with an acute exacerbation of chronic pancreatitis. He died as a result of multi-organ failure after a leak developed at the site of his cholecystojejunal anastomosis. Warshaw *et al.*[5] found similarly pleasing results following surgical intervention and suggested that the patient's pain in this particular subset of patients was due to biliary as distinct from pancreatic obstruction. Other authors[13,15,29,30,31], however, found that pain was not improved after biliary bypass. Since the minimum time of follow-up in this series of patients has been two years, we would suggest that the improvement in pain is probably clinically significant[32]. It is important that both physicians and surgeons dealing with pancreatitis and dealing with obstructive jaundice remain aware of the association between the two conditions. Jaundice due to biliary obstruction by benign pancreatic pathology is easily overlooked but remains a remediable cause of symptoms and complications in patients with chronic pancreatitis. It is also important to realise that clinical jaundice due to chronic pancreatitis can present as relatively painless jaundice, although it is more common to see jaundice in a patient with long-standing chronic pancreatitis. Stenting should probably be avoided in such patients. The general prognosis seems to be good for jaundice, for pain and for life. Chronic pancreatitis may constitute yet another "malignant masquerade"[33] in jaundiced patients.

### *References*

1. Sarles, H. and Sahel, J. (1978). Cholestasis and lesions of the biliary tract in chronic pancreatitis. *Gut*, **19**, 851–7.
2. Scott, J., Summerfield, J.A., Elias, E., Dick, R. and Sherlock, S. (1977) Chronic pancreatitis: a cause of cholestasis. *Gut*, **18**, 196–201.

3. Bradley, E.L., Salam, A.A. (1979). Hyperbilirubinaemia in inflammatory pancreatic disease. *Annals of Surgery*, **188**, 629–9.
4. Afroudakis, A., Kaplowitz, N. (1981) Liver histopathology in chronic common bile duct stenosis due to chronic alcoholic pancreatitis. *Hepatology*, **1**, 65-72.
5. Warshaw, A.L., Schapiro, R.H., Ferrucci, J.T., Goldabini, J.J. (1976) Persistent obstructive jaundice, cholangitis and biliary cirrhosis due to common bile duct stenosis in chronic pancreatitis. *Gastroenterology*, **70**, 562-7.
6. Harris, A.I., Korsten, M.A. (1976) Acute suppurative cholangitis secondary to calcific pancreatitis. *Gastroenterology*, **71**, 847-50.
7. Snape, W.J., Long, W.B., Trotman, B.W., Marin, G.A., Czaja, A.J. (1976) Marked alkaline phosphatase elevation with partial common bile duct obstruction due to calcific pancreatitis. *Gastroenterology*, **70**, 70-3.
8. Yadegar, J., Williams, R.A., Passaro, E., Wilson, S.E. (1980) Common duct stricture from chronic pancreatitis. *Archives of Surgery*, **5**, 582-6.
9. Segal, I., Lawson, H.H., Rabinowitz, B., Hamilton, D.G. (1982) Chronic pancreatitis and the hepatobiliary system. *American Journal of Gastroenterology*, **77**, 867-74.
10. Gullo, L., Costa, P.L., Labo, G. (1977) Chronic pancreatitis in Italy. Etiological, clinical and histological observations based on 253 cases. *Rendiconti Gastroenterologie*, **9**, 97-104.
11. Aranha, G.V., Prinz, R.A., Freeark, R.J., Greenlee, H.B. (1984) The spectrum of biliary tract obstruction from chronic pancreatitis. *Archives of Surgery*, **119**, 595-600.
12. Warshaw, A.L. (1985) Conservation of pancreatic tissue by combined gastric, biliary, and pancreatic duct drainage for pain from chronic pancreatitis. *American Journal of Surgery*, **145**, 563-8.
13. Juttner, H.U., Renner, I.G., Richman, R.H., Redeker, A.G. (1976) Evaluation of obstructive jaundice in chronic pancreatitis. *Gastroenterology*, **70**, A40/898.
14. Littenberg, A., Afroudakis, A., Kaplowitz, N. (1979) Common bile duct stenosis from chronic pancreatitis: A clinical and pathologic spectrum. *Medicine*, **58**, 385-412.
15. Newton, B.B., Rittenbury, M.S., Anderson, M.C. (1983) Extrahepatic biliary obstruction associated with pancreatitis. *Annals of Surgery*, **197**, 645-53.
16. Shi, E.C.P., Ham, J.M. (1980) Benign biliary strictures associated with chronic pancreatitis and gallstones. *Australian and New Zealand Journal of Strategy*, **50**, 488-92.
17. Fraser, J. (1938) The surgical treatment of obstructive jaundice in pancreatic disease. *British Journal of Strategy*, **26**, 393-411.
18. Mayo Robson, A.W. (1900) Pancreatitis with especial reference to chronic pancreatitis. *Lancet*, **2**, 235-40.
19. Mikulicz, Baron von. (1903) Surgery of the pancreas. *Annals of Surgery*, **38** 1-29.
20. Rowlands, R.P. (1910) Jaundice due to chronic pancreatitis. *Guys Hosp. Gazette*, **24**, 269–72.
21. Lorelius, L.E., Jacobson, G., Sawada, S. (1982) Endoprosthesis as an internal biliary drainage in inoperable patients with biliary obstruction. *Acta Chirurgia Scandinavica*, **148**, 613-6.
22. Tytgat, G.N.J., Huibregtse, K., Bartelsman, J.F.W.M., Den Hartog Jager, F.C.A. (1986) Endoscopic palliative therapy of gastrointestinal and biliary tumours with prostheses. *Clinics in Gastroenterology*, **15**, 249-71.
23. Beazley, R.M. (1981) Needle biopsy diagnosis of pancreatic cancer. *Cancer*, **47**, 1685-7.
24. Evander, A., Ihse, I., Lunderguist, A., Tylen, U., Akerman, M. (1978) Percutaneous cytodiagnosis of carcinoma of the pancreas and bile duct. *Annals of Surgery*. **188**, 90-2.
25. Hewett, P.S., Langlois, S.L., Orell, S.R. (1988) The diagnosis of mass lesions in the pancreas by fine needle aspiration biopsy. *Journal of Gastroenterology and Hepatology*, **3**, 71-6.
26. Kraus, M.A., Wilson, S.D., (1980) Choledochoduodenostomy: importance of common duct size and occurrence of cholangitis. *Archives of Surgery*, **115**, 1212-3.
27. Gregg, J.A., Carr-Locke, D.L., Gallagher, M.M., (1981) Importance of common bile duct stricture associated with chronic pancreatitis. Diagnosis by endoscopic retrograde cholangiopancreatography. *American Journal of Surgery*, **141**, 199-203.
28. Schulte, W.J., LaPorta, A.J., Condon, R.E., Unger, G.F., Geenen, G.E., DeCosse, J.J. (1977) Chronic pancreatitis: a cause of biliary stricture. *Surgery*, **82**, 303-9.
29. Brinton, M.H., Pellegrini, C.A., Stein, S.F., Way, L.W. (1984) Surgical treatment of chronic pancreatitis. *American Journal of Surgery*, **148**, 754-9.
30. Creaghe, S.B., Rosenman, D.M., Sark, R.P. (1981) Biliary obstruction in chronic pancreatitis: indications for surgical intervention. *American Journal of Surgery*, **47**, 243-6.
31. Way, L.W., Gadacz, T., Goldman, L. (1974) Surgical treatment of chronic pancreatitis. *American Journal of Surgery*, **127**, 202-9.

32. Little, J.M. (1983). Chronic pancreatitis: results of a protocol of management. *Australian and New Zealand Journal of Surgery,* **53,** 403–9.
33. Hadjis, N.S., Collier, N.A., Blumgart, L.H. (1985). Malignant masquerade at the hilum of the liver. *British Journal of Surgery,* **72,** 659–61.

*Accepted by S. Bengmark on 6 November 1988*

